# Genetic basis of osteogenesis imperfecta from a single tertiary centre in South Africa

**DOI:** 10.1038/s41431-023-01509-3

**Published:** 2023-12-15

**Authors:** Kimberly Christine Coetzer, Ekkehard Zöllner, Shahida Moosa

**Affiliations:** 1 Division of Molecular Biology and Human Genetics, Stellenbosch University Faculty of Medicine, and Health Sciences, Tygerberg, 7505 Cape Town, South Africa; 2 Department of Paediatrics, Stellenbosch University Faculty of Medicine, and Health Sciences, Tygerberg, 7505 Cape Town, South Africa; 3 Medical Genetics, Tygerberg Hospital, Tygerberg, 7505 Cape Town, South Africa

**Keywords:** Genetics research, Endocrine system and metabolic diseases

## Abstract

Osteogenesis imperfecta (OI) is a clinically heterogeneous disorder characterised by skeletal fragility and an increased fracture incidence. It occurs in approximately one in every 15–20,000 births and is known to vary considerably in its severity. This report aimed to use next-generation sequencing (NGS) technology to identify disease genes and causal variants in South African patients with clinical-radiological features of OI. A total of 50 affected individuals were recruited at Tygerberg Hospital’s Medical Genetics clinic. Patients were selected for a gene panel test (*n* = 39), a single variant test (*n* = 1) or exome sequencing (ES) (*n* = 12, 7 singletons, 1 affected duo, and 1 trio), depending on funding eligibility. An in-house genomic bioinformatics pipeline was developed for the ES samples using open-source software and tools. This study’s 100% diagnostic yield was largely attributable to an accurate clinical diagnosis. A causal variant in *COL1A1* or *COL1A2* was identified in 94% of this patient cohort, which is in line with previous studies. Interestingly, this study was the first to identify the common South African pathogenic *FKBP10* variant in a patient of mixed ancestry, adding to what was previously known about this variant in our population. Additionally, a recurrent variant in *COL1A2*: c.1892G>T was discovered in 27 individuals (25 from three large unrelated families and two further individuals), facilitating the establishment of local testing for this variant in South African patients.

## Introduction

Osteogenesis imperfecta (OI) (OMIM 166200), or brittle bone disease, is one of the more common skeletal dysplasias, that occurs in approximately one in 15–20,000 births [1]; however, the exact prevalence in South African populations is still unknown [2]. It has a heterogenous clinical presentation characterised by skeletal fragility and an increased fracture occurrence with variable severity. Additional clinical features can include, among others, blue sclerae, hearing loss, hyperlaxity of the joints and skin, dentinogenesis imperfecta (DI), Wormian bones, scoliosis, and short stature [3], depending on the subtype.

Knowledge of osteogenesis imperfecta’s (OI) genetic and molecular heterogeneity has increased exponentially since the introduction of next-generation sequencing (NGS) technology in recent years [4], resulting in the identification of 20 genes which have been shown to underlie OI according to OMIM (https://www.omim.org/). However, pathogenic variants in *COL1A1* (OMIM 120150) and *COL1A2* (OMIM 120160) are responsible for the majority of cases (~90%) [5]. Through the discovery of novel OI-causing genes, it was found that defects in collagen production, structure, processing, posttranslational modification, folding, and crosslinking can all result in OI as well as deficits in osteoblasts and bone mineralisation [6].

Since the introduction of NGS technology into diagnostics, a wide range of molecular diagnostic procedures, such as single-gene or variant tests, gene panel tests, and exome/genome sequencing (ES/GS) have become available. Despite significant progress in NGS approaches, it still takes an average of 8 years to get an accurate diagnosis for rare diseases with some patients taking up to 30 years to reach a diagnosis [7].

This research sought to identify disease genes and causal variants in patients with clinical-radiological features of osteogenesis imperfecta (OI) in a South African tertiary institute in an effort to understand the underlying molecular genetic causes of OI in our understudied population.

## Materials and methods

### Patient recruitment

Individuals suspected of having osteogenesis imperfecta (OI) were recruited at Tygerberg Hospital’s (TBH) Medical Genetics clinic, including 47 singletons, one duo of affected mother and child and one trio with two healthy parents. These individuals were assessed, and full family and medical histories, as well as information on past tests, were obtained. All patients/parents signed informed consent for testing and inclusion in this study (SU HREC Ref N18/03/031). Patients were assigned a randomly generated code to preserve anonymity. Patients who presented with clinical signs of OI (Supplementary Table 1) and no molecular diagnosis were eligible for the study. The final patient cohort had the following tests performed: (1) ES [singleton exome (*n* = 7), duo exome with an affected mother and child (*n* = 2, patients MXHC and MXHCM), a trio exome with unaffected parents (*n* = 1, patient KTWW). Patients KTWW and MXHC’s parents also provided informed consent for secondary findings to be reported back to them; (2) a gene panel test (*n* = 39) and (3) an *FKBP10* single variant test (*n* = 1). Test selection was based on funding eligibility. ES was conducted within the Undiagnosed Disease Programme (UDP) [8].

### Sample collection, extraction, and exome sequencing

Following informed consent, saliva or blood samples were obtained from each patient. Saliva samples were sent directly for an OI gene panel (*n* = 39; Invitae, USA) or Sanger sequencing of a common *FKBP10* variant test (*n* = 1), respectively. For the remaining samples (*n* = 12), DNA was extracted using standard protocols (Supplementary 1). Each sample was given a unique code to ensure patient confidentiality and anonymity. Exome sequencing was performed as previously described [8].

### Bioinformatics analysis: ES data

For the ES samples included in this study, we employed an in-house pipeline for variant calling, filtering, annotation, and prioritisation which made use of readily available open-source software and tools as previously described [8]. In brief, our in-house pipeline included variant annotation using Annovar’s filter-based approach (https://annovar.openbioinformatics.org/en/latest/), population and quality filtering, prioritisation using Exomiser (V.12.1.0., available at https://github.com/exomiser/Exomiser), literature interpretation, and ACMG/AMP classification [9]. Tolerated and benign variants were removed based on pathogenicity scores and already-published data on ClinVar (https://www.ncbi.nlm.nih.gov/clinvar/), Genome Aggregation Database (gnomAD; https://gnomad.broadinstitute.org/), the Leiden Open Variation Database (LOVD; https://www.lovd.nl/) and Varsome (https://varsome.com/). Candidate variants for each patient were inspected further on Integrative Genomics Viewer (IGV; https://software.broadinstitute.org/software/igv/). The final likely causative variant identified through this in-house pipeline was manually classified using ACMG/AMP guidelines [9]. A variant validator (https://variantvalidator.org/) was employed to confirm and validate the variant names.

### Gene panel test and *FKBP10* single variant test

The analysis for the gene panels was performed at Invitae (USA) and the *FKBP10* common variant at the NHLS laboratory at the University of Cape Town, South Africa. Patient samples (*n* = 39) were sent for an Osteogenesis Imperfecta and Bone Fragility Panel (Invitae, USA), which consisted of 67 genes related to OI and bone fragility. This comprised 18 of the 20 OI genes that have already been identified [panel excluded *KDELR2* (OMIM 609024) and *CCDC134* (OMIM 618788)]. The variants identified through this panel formed part of a growing in-house OI variant database. Diagnostic Sanger sequencing of a single *FKBP10* variant known to be common in OI patients in our population (*FKBP10*:c.831dup) [10] was performed for one patient (Patient 14).

## Results

### Patient demographics and clinical diagnoses

The study enrolled 52 South Africans, consisting of 50 individuals suspected of having osteogenesis imperfecta (OI) and the healthy parents of patient KTWW. The age of affected patients at the time of testing varied from stillbirth to 37 years. In all, 33/50 (66%) patients were female, and 17/50 (34%) were male (Table 1).

**Table 1:** Results from ES, multi-gene panel and single variant test.

Patient ID^a^	Gene: variant	ACMG/AMP classification	ClinVar accession	Test performed
CGFB	*COL1A1*:c.2596G>A,p.Gly866Ser	P (PM1, PM2, PP2, PP3, PP4, PS1)	VCV000425612.14	ES
CUOV	*COL1A2*:c.1927G>A,p.Gly643Arg^b^	LP (PM1, PM2, PP2, PP3, PP4)	Not reported	ES
DUQR	*COL1A2:*c.1892G>T,p.Gly631Val	P (PM1, PM2, PP2, PP3, PP4, PS1)	VCV000663292.10	ES
GDQI	*COL1A1*:c.1082_1099dupGAGGCTCTGAAGGTCCCC,p.Arg361_Pro366dup	LP (PM1, PM2, PM4, PP4)	VCV001333640.1	ES
KTWW	*COL1A2*:c.1378G>A,p.Gly460Ser also, BRCA1: c.181T>G,p.Cys61Gly	P (PM1, PM2, PP2, PP3, PP4, PS1, PS2), P (PM1, PM2, PP2, PP3, PS1, BP1)	VCV000526895.13, VCV000017661.105	ES
MXHC	*COL1A1*:c.2775delT,p.Gly926Valfs*182 also, *PTPN11*:c.1492C>T,p.Arg498Trp	P (PVS1, PM2, PP1, PP4, PS1), P (PM1, PM2, PP2, PP3, PS1)	VCV000236248.7, VCV000040553.48	ES
MXHCM	*COL1A1*:c.2775delT,p.Gly926Valfs*182	P (PVS1, PM2, PP1, PP4, PS1)	VCV000236248.7	ES
PXYU	*IFITM5*:c.-14C>T	P (PM2, PM4, PP1, PP4, PS1, PS3)	VCV000037143.33	ES
SWFC	*COL1A1*:c.1292G>T,p.Gly431Val	LP (PM1, PM2, PVS1, PP1, PS1)	RCV001808843.1	ES
YMUQ	*COL1A1*:c.1049G>T,p.Gly350Val^b^	LP (PM1, PM2, PP2, PP3, PP4)	Not reported	ES
F1.1–F1.6 (*n* = 6)	*COL1A2*:c.1892G>T,p.Gly631Val	P (PS1, PM1, PM2, PP2, PP4)	VCV000663292.10	Gene panel
F2.1–F2.9 (*n* = 9)	*COL1A2*:c.1892G>T,p.Gly631Val	P (PS1, PM1, PM2, PP2, PP4)	VCV000663292.10	Gene panel
F3.1–F3.10 (*n* = 10)	*COL1A2*:c.1892G>T,p.Gly631Val	P (PS1, PM1, PM2, PP2, PP4)	VCV000663292.10	Gene panel
P1	*COL1A1*:c.458_459insN[72],p.Gly16Leu155insN[24]^b^	P (PM1, PM2, PP3, PP4)	Not reported	Gene panel
P2	*COL1A1*:c.4163T>C,p.Leu1388Pro	LP (PM2, PS1, PP3, PP4)	RCV001044363.4	Gene panel
P3	*COL1A2*:c.1459G>A,p.Gly487Arg	P (PM1, PM2, PP2, PP4, PS1)	VCV000947920.6	Gene panel
P4	*COL1A2*:c.2215G>A,p.Gly739Arg	P (PM1, PM2, PP2, PP4, PS1)	VCV000934873.1	Gene panel
P5	*COL1A2*:c.2341G>C,p.Gly781Arg	P (PM1, PM2, PP2, PP4, PS1)	VCV000841344.6	Gene panel
P6	*COL1A2*:c.767G>A,p.Gly256Asp	P (PM1, PM2, PP2, PP4, PS1)	RCV001222603.2	Gene panel
P7	*COL1A1*:c.458_459insN[72],p.Gly16Leu155insN[24]^b^	P (PM1, PM2, PP3, PP4)	Not reported	Gene panel
P8	*COL1A2*:c.1028G>A,p.Gly343Glu^b^	P (PM1, PM2, PP3, PP4)	Not reported	Gene panel
P9	*COL1A1*:c.750+1G>T^b^	P (PM2, PM5, PP3, PP4, PVS1)	Not reported	Gene panel
P10	*COL1A2*:c.2314G>A,p.Gly772Ser	P (PP5, PM5, PP3, PM1, PM2)	VCV000420022.25	Gene panel
P11	*COL1A2*:c.2314G>C,p.Gly772Arg	P (PM5, PP3, PM1, PP5, PM2)	VCV001468991.4	Gene panel
P12	*COL1A1*:c.2830-2A>C^b^	P (PM2, PM5, PP3, PP4, PVS1)	Not reported	Gene panel
P13	*FKBP10*:c.831dupC	P (PM2, PM4, PP4, PS1, PS3)	VCV000438659.48	Gene panel
P14	*FKBP10*:c.831dupC	P (PM2, PM4, PP4, PS1, PS3)	VCV000438659.48	Single variant test
P15	*COL1A2*: c.1892G>T,p.Gly631Val	P (PS1, PM1, PM2, PP2, PP4)	VCV000663292.10	Gene panel

*P* pathogenic, *LP* likely pathogenic, *VUS* variant of uncertain significance, *F* family, *P#* patient number, *ES* exome sequencing.

^a^The WES patient ID codes are randomly generated and are completely anonymised.

^b^Novel variant.

### Exome sequencing

All ten ES patients were successfully diagnosed with OI using the in-house pipeline and were manually classified as “pathogenic” (*n* = 6) or “likely pathogenic” (*n* = 4) using ACMG/AMP guidelines [9] (Supplementary Fig. 1). Eight patients had known pathogenic/likely pathogenic OI-causing variants, while two patients (CUOV and YMUQ) had a novel likely pathogenic OI-causing variant (Table 1). These variants have since been reported by the Undiagnosed Disease Program (UDP) [8].

Six out of the ten (60%) affected patients were found to have disease-causing variants in *COL1A1*. These patients have been clinically diagnosed with perinatal lethal OI (SWFC and YMUQ), progressively deforming OI (GDQI), and moderate OI (CGFB and MXHC/M) (Supplementary Table 1). Patient SWFC was stillborn while patient YMUQ was predicted to be perinatal lethal but has survived with very severe OI and early deformities. An additional three patients (30%) had variants in *COL1A2*. These are patients KTWW, DUQR, and CUOV who have been clinically diagnosed with perinatal lethal, moderate, and progressively deforming OI, respectively (Supplementary Table 1). Patient KTWW also survived but has very severe OI and early deformities. This resulted in 90% of the ES patients in this study having a variant in either *COL1A1* or *COL1A2* (Supplementary Fig. 2). The remaining ES patient had a common recurrent variant in *IFITM5* (c.-14C>T) (Table 1).

### Secondary findings

The parents of patients KTWW and MXHC gave informed consent for secondary findings. A previously reported pathogenic variant was found in *BRCA1* (OMIM 113705) (c.181T>C; ClinVar VCV000017661.97) in patient KTWW, which is implicated in breast and ovarian cancer (OMIM 604370). Further analysis showed that this variant was inherited from his healthy father (KTWWF). The second patient, MXHC, was found to have a previously reported pathogenic variant in *PTPN11* (OMIM 176876) (c.1492C>T; ClinVar VCV000040553.46), which is implicated in Noonan Syndrome (OMIM 163950). Both of these variants were classified as “pathogenic” according to ACMG/AMP guidelines [9].

### Gene panel test/single-gene test

Patient samples (*n* = 39) were sent for a multi-gene panel test. Through this test, it was found that 26 patients from four unrelated families (F1, F2, F3 and P15) shared the same pathogenic variant in *COL1A2* (c.1892G>T; ClinVar VCV000663292.10) (Table 1). Overall ~95% (37/39) of patients had a variant in *COL1A1* or *COL1A2*. One patient (P14) had Sanger sequencing for a common *FKBP10* variant (c.831dup; ClinVar VCV000438659.48). This variant was also identified in patient 13, who had a gene panel. Three novel pathogenic variants were identified in *COL1A1* in patient 12 (c.2830-2A>C), patient 7 (c.458_459insN[72]) and patient 9 (c.750+1G>T). A fourth novel variant was detected in patient 8 (*COL1A2*:c.1028G>A). These variants have not been previously reported on ClinVar or LOVD (Table 1).

**Fig. 1:**
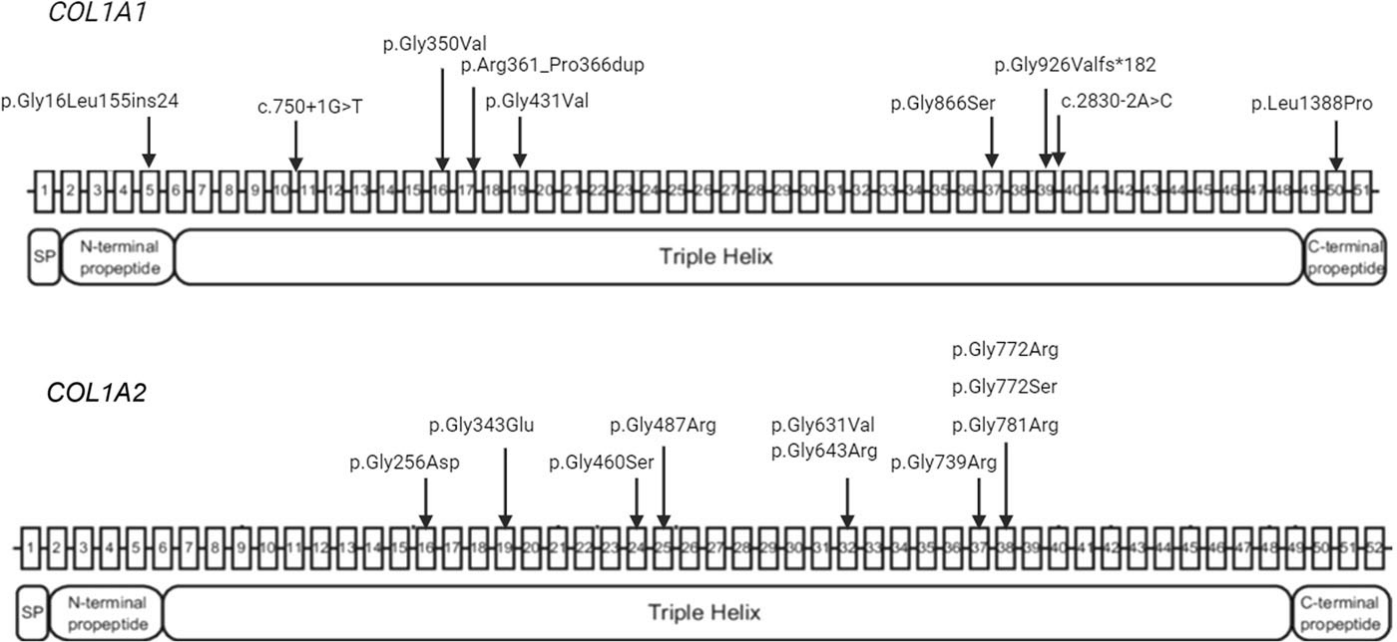
Graphical representation of *COL1A1* and *COL1A2* genes showing the variants identified. Mutational spectrum of variants on *COL1A1* and *COL1A2* genes.

Finally, the data collected from this study revealed that nine unique P/LP variants were identified in *COL1A2* as well as 1 recurring variant, which was identified in 27 patients indicating a possible mutational hotpot in this region (Fig. 1). Conversely, seven unique variants were detected in the *COL1A1* gene, with two recurring variants present in two patients each. Six variants are reported here for the first time: four in *COL1A1* and two in *COL1A2* (Table 1).

## Discussion

We used ES, gene panel testing, and a single variant test to achieve a diagnostic yield of 100% for patients with OI in this study. The patient demographic is representative of the Western Cape Province, with Tygerberg Hospital being the largest tertiary referral centre in the province. Since it is part of a paediatric clinic, the majority of the patients are under 18 years of age.

As previously stated, research indicates that pathogenic variants in the *COL1A1* or *COL1A2* genes account for approximately 90% of OI cases [5]. This was corroborated by this study, which observed that ~94% of the patients in this cohort had a likely causal variant in *COL1A1* or *COL1A2*, proving that variants in these genes underlie the majority of OI in our population.

Some patients of note in this study are patients YMUQ and KTWW. Based on their identified variants, it was expected that patients SWFC and YMUQ would have a lethal phenotype, given that they both have *COL1A1* Gly-to-Val substitutions towards the C-terminal of residue 200, which is almost always lethal [11]. However, YMUQ survived with severe deformities, while SWFC succumbed to the condition at age 2 years. Patient KTWW also survived but with severe deformities, in line with the lethal cluster in the α2(I) chain, where their variant was located [11]. It is worth noting that the phenotypes of smaller side-chain residues, such as serine, are more variable and can impact the stability profile of the triple helix. This variability is reflected in the severity of OI type 2 in patients like KTWW. This supports research showing that each chain has its own genotype–phenotype relationship, supporting the notion of a regional model of OI pathophysiology. Phenotypic variability in OI can be caused by the affected collagen chain gene, the type and location of the variant, the chemical nature of the substituting amino acid, or a combination [11, 12].

The common variant (*COL1A2*:c.1892G>T) identified in 27 (*n* = 1 ES (DUQR) and *n* = 26 gene panel) patients is an example of a non-lethal valine substitution in *COL1A2* (p.Gly631Val) (Table 1). Gly-to-Val substitutions in α2(1) were found to be more variable than in α1(I), explaining these patients’ milder phenotype [11]. We suggest that this is the first report of this variant in an African population. All patients and families are of mixed South African ancestry and further research is needed to explore if they share a common haplotype.

**Fig. 2:**
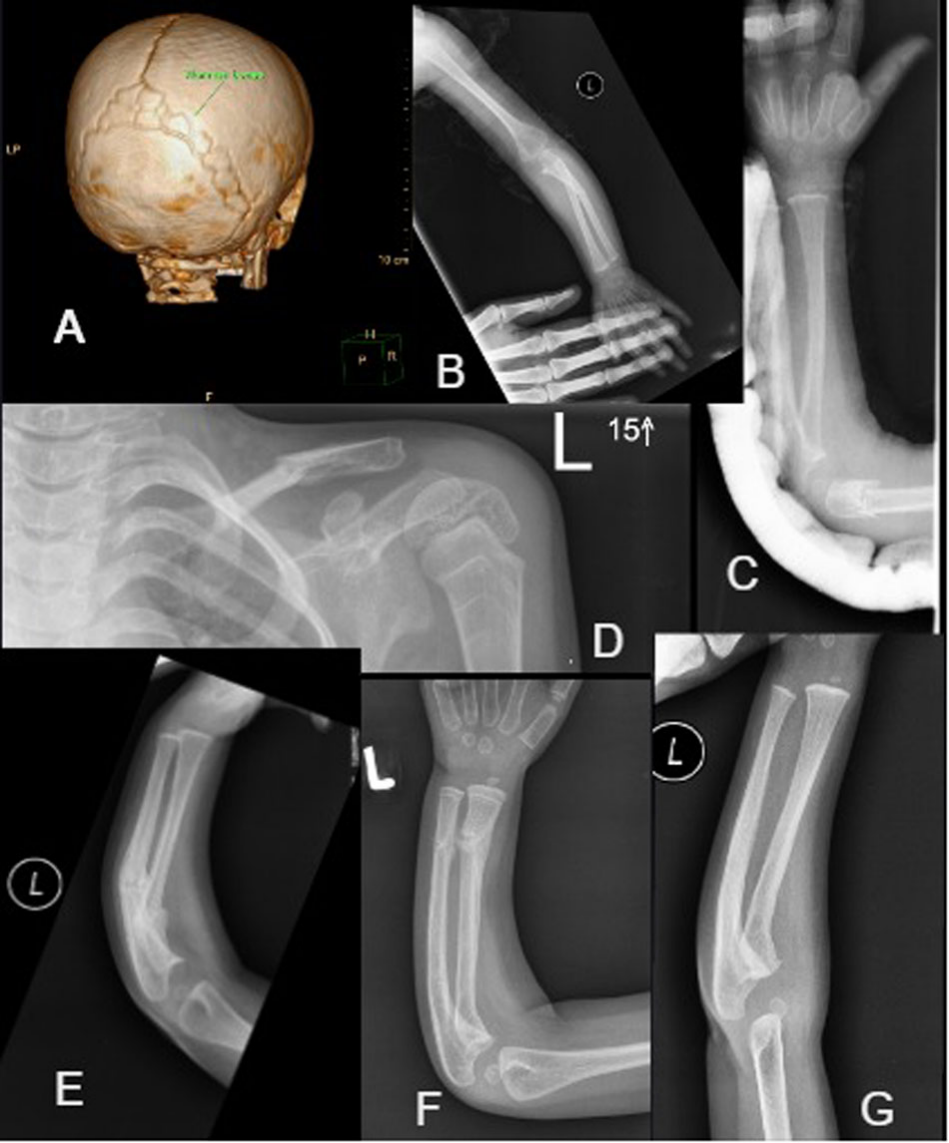
Radiographs of patient PXYU (*IFITM5*). **A** CT skull (age 2.5 years): showing numerous Wormian bones. **B** Fracture left ulna (midshaft) at 13 months—note no obvious callous formation. **C** Fracture distal ulna and radius at 23 months. **D** Fracture left clavicle at age 4 years (note zebra stripes on proximal humerus from bisphosphonate therapy). **E** Fracture midshaft ulna and radius at 14 months with mild callus formation. **F** & **G** Healed proximal ulna/radius fracture at 19 months.

While less common, non-collagen genes can harbour OI-causing variants as seen in 3 patients (PXYU, P13 and P14). Patient PXYU was found to have a heterozygous pathogenic frameshift variant in *IFITM5* (c.-14C>T; ClinVar VCV000037143.35) and was clinically diagnosed with a progressively deforming type of OI (Fig. 2). Based on prior studies, this variant has been shown to be pathogenic and results in OI type V (OMIM 610967) in 5–10% of all OI cases [13]. Clinical features frequently associated with OI type V include a propensity to form hyperplastic calluses following fractures, calcification of the interosseous membrane of the forearm, and a radiodense metaphyseal line of the long bones [14]. Interestingly, this patient did not display any of the typical radiological features of OI Type V on plain film X-ray. She was 5 years old already, had sustained several fractures, and was wheelchair-bound. High phenotypic variability is common in individuals with OI type V as evidenced In this patient, presumably due to modifier genes [15].

**Fig. 3:**
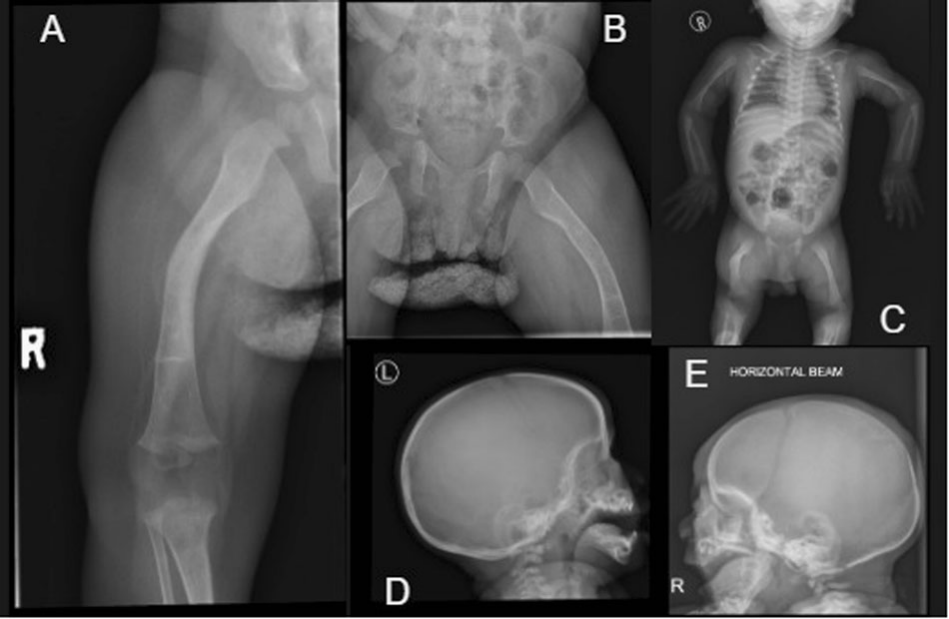
Radiographs of patient 13 (*FKBP10*). **A** Right femur deformity with osteopenia, metaphyseal flaring at the knee at age 13 months. **B** Right femur showing deformity, osteopenia at age 11 months. **C** Babygram at age 9 days showing generalised osteopenia and angulated right femur. **D** Lateral skull at 11 months showing Wormian bones. **E** Lateral skull at 9 days showing Wormian bones and patent coronal sutures.

**Fig. 4:**
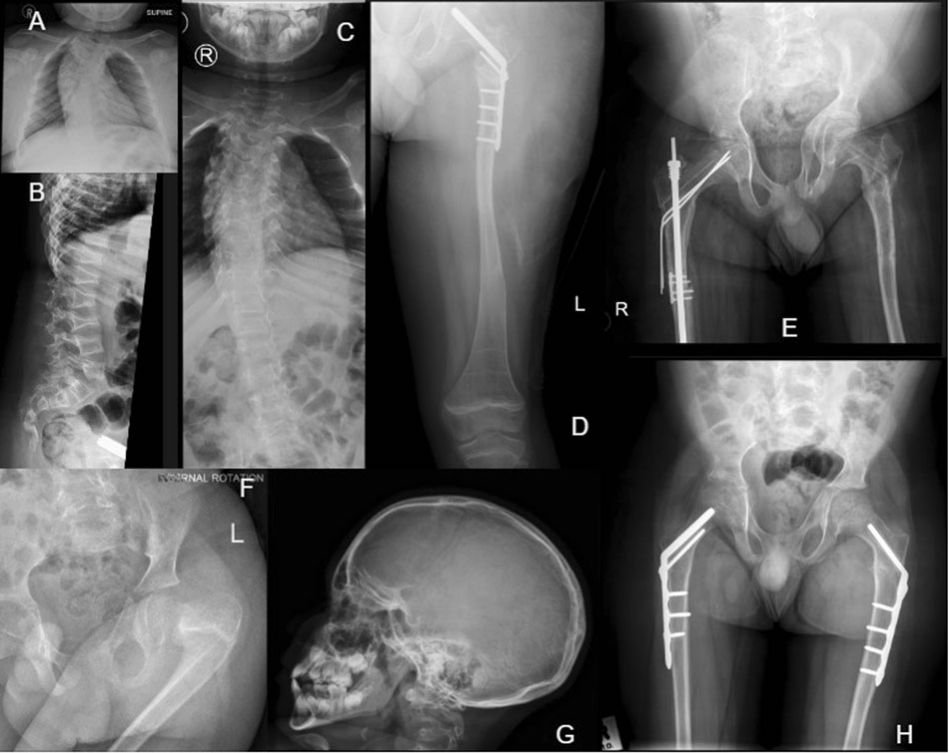
Radiographs of patient 14 (*FKBP10*). **A** AP chest showing scoliosis at age 9 years. **B** Lateral spine showing wedge fractures of the lumbar vertebrae at age 9 years. **C** AP chest showing scoliosis and abnormal vertebrae at age 10 years. **D** Left leg showing mild deformity, thin femoral shafts, zebra stripes from bisphosphonate therapy, and orthopaedic nailing of femoral head at age 12 years. **E** AP pelvis and proximal femora showing asymmetrical pelvis, thin femoral shafts, osteopenia and rodding of the right femur at age 13 years. **F** Fracture and deformity of left femoral head at age 5 years. **G** Lateral skull showing numerous Wormian bones at age 9 years. **H** AP pelvis and proximal femora with corrective orthopaedic nails and plates at age 7 years.

Patients 13 and 14 were found to share an identical homozygous *FKBP10* variant (c.831dup), which is a common South African variant known to cause progressively deforming OI. Both had multiple fractures in infancy and childhood, dentinogenesis imperfecta and greyish sclerae. Patient 13 is more severely affected, has severe deformities and is wheelchair-bound (Fig. 3). He is the only affected person in his family. Patient 14 has undergone multiple orthopaedic interventions and mobilises with assistive devices (Fig. 4). The significantly high occurrence of this variant in South African populations implies that it is the result of a common ancestral founder effect in the Indigenous Black African population group [8]. As Patient 14 presented with typical FKBP10-related OI, we chose to test for the common variant first. This testing is available through our National Health Laboratory Service and is often first-line testing for Black South African patients presenting with progressively deforming OI. Interestingly, in this study, this variant was identified for the first time in a patient of mixed South African ancestry (Patient 13), adding to what we previously knew about this specific variant in our population. An implication of this might be to include more patients in testing for this variant, not only Black African patients, as is current practice.

A pathogenic variant in *BRCA1* (c.181T>G) known to cause a predisposition to inherited breast and ovarian cancer (OMIM 604370) was identified in patient KTWW. Thus far, only two further family members have presented for genetic counselling and testing. Both have been enrolled in appropriate cancer surveillance programmes. A pathogenic variant in *PTPN11* (c.1492C>T) known to be implicated in Noonan syndrome (OMIM 601321) was identified in patient MXHC. Reverse phenotyping revealed that the patient had some features in keeping with Noonan syndrome. Thus, she has a dual diagnosis. While *PTPN11* is not part of the 73 actionable genes, it was decided by the medical geneticist to report this variant back to the family, as it has important clinical and reproductive implications.

## Conclusion

Several genetic testing strategies can be employed to end a patient’s diagnostic odyssey. Multi-gene panels, such as those performed on the 39 patients in this study, are limited in that they can quickly become “out of date” as new genes are discovered. This is a significant disadvantage, as evidenced by Invitae’s Osteogenesis Imperfecta and Bone Fragility Panel, which contains only 18 of the 20 known OI genes, excluding *KDELR2* (OMIM 609024) and *CCDC134* (OMIM 618788), both of which were only discovered in 2020 [16, 17]. Updating multi-gene panels is an arduous task that is done too infrequently, slowing the diagnosis of patients with rare monogenic diseases. As a result, alternative methods for avoiding this were required. The use of ES supplemented with virtual gene panels in this study was one way to address this issue. Regardless of the test used, all 50 (100%) OI-affected patients were diagnosed, and both the multi-gene panel test and ES were able to identify novel OI-causing variants. Overall, ES supplemented with virtual gene panels appears to be a more suitable option than single variant and multi-gene panel testing because it can be easily tailored to each case.

Finally, local classification has the advantage of allowing prior knowledge of ethnicity, other data like in-house variant databases, knowledge about segregation in the family, and de novo status of the variant(s) to be integrated. This is beneficial in our understudied population since some variants are known to be relatively frequent in specific populations in South Africa. An example of this is a variant in *FKBP10* (c.831dup) which is common in the Black African population.

This study showed that even in an understudied African population, multi-gene panels and especially ES can be successfully used in genetically heterogeneous conditions such as OI. ES allowed further information to be delivered to the families (secondary findings and a dual diagnosis), which would have been missed on a targeted panel. Recurrent variants form the basis of further study and can be easily translated into cost-effective local testing.

## Supplementary information

Table S1

Supplementary information

## Data Availability

The variants have been submitted to ClinVar—all accession numbers are available in Table 1. Previously unreported variants have been submitted to ClinVar (accession numbers SCV004101794–SCV004101798). All data are available on request.
