# Supplementary material for: Genetic basis of osteogenesis imperfecta from a single tertiary centre in South Africa
Source: Eur J Hum Genet. 2023 Dec 15;32(10):1285–90. doi: 10.1038/s41431-023-01509-3 (PMC11499597; doi:10.1038/s41431-023-01509-3)
Supplement: Supplementary file 1 — Table S1 [file 41431_2023_1509_MOESM1_ESM.docx]

**Table S1.** Clinical features of patients who underwent whole exome sequencing (CGFB - YMUQ), gene panels (F1.1 - P15)  and a single variant test (P14)

| Patient ID | Gene:Variant | Age at test | Ancestry | Gender | Clinical Type | Mobility | Short stature | Sclerae | DI | HL | Osteopenia | Frequent fractures | Secondary findings consent |
| --- | --- | --- | --- | --- | --- | --- | --- | --- | --- | --- | --- | --- | --- |
| CGFB | *COL1A1*:c.2596G>A,p.Gly866Ser | 5 years 2 months | Black South African | F | Type 4 | walking | yes | grey | yes | no | yes | yes | No |
| CUOV | *COL1A2*:c.1927G>A,p.Gly643Arg(*) | 15 years | Black South African | F | Type 3 | Walking - with assistive device | yes | blue | yes | no | yes | yes | No |
| DUQR | *COL1A2*:c.1892G>T,p.Gly631Val | 1 year | Mixed South African | F | Type 4 | N/A | yes | grey | yes | no | yes | yes | No |
| GDQI | *COL1A1*:c.1082_1099dupGAGGCTCTGAAGGTCCCC,p.Arg361_Pro366dup | 12 years | Mixed South African | M | Type 3 | wheelchair-bound | yes | blue | yes | no | yes | yes | No |
| KTWW | *COL1A2*:c.1378G>A,p.Gly460Ser also, BRCA1: c.181T>G,p.Cys61Gly | 6 months | Mixed South African | M | Type 3 | never sat, mobilises with a buggy | yes | blue | yes | no | yes | yes – multiple in-utero | Yes |
| KTWWF (healthy father) | BRCA1: c.181T>G,p.Cys61Gly | 29 years | Mixed South African | M | Healthy | N/A | N/A | N/A | N/A | N/A | N/A | N/A | Yes |
| KTWWM (healthy mother) | None | 25 years | Mixed South African | F | Healthy | N/A | N/A | N/A | N/A | N/A | N/A | N/A | Yes |
| MXHC | *COL1A1*:c.2775delT,p.Gly926Valfs*182 also, PTPN11:c.1492C>T,p.Arg498Trp | 6 years | Mixed South African | F | Type 4 | walking | yes | grey | yes | no | yes | yes | Yes |
| MXHCM (affected mother) | *COL1A1*:c.2775delT,p.Gly926Valfs*182 | 37 years | Mixed South African | F | Type 4 | walking | yes | grey | yes | no | yes | yes | Yes |
| PXYU | *IFITM5*:c.-14C>T | 5 years | Black South African | F | Type 3 | wheelchair-bound | yes | blue | yes | no | yes | yes | No |
| SWFC | *COL1A1*:c.1292G>T,p.Gly431Val | Stillborn | Black South African | M | Type 2 | N/A | yes | blue | yes | no | yes | yes – multiple in-utero | No |
| YMUQ | *COL1A1*:c.1049G>T,p.Gly350Val(*) | 4 months | Mixed South African | M | Type 3 | never sat, mobilises with a buggy | yes | blue | yes | no | yes | yes – multiple in-utero | No |
| F1.1 | *COL1A2*:c.1892G>T,p.Gly631Val | 28 years | Mixed South African | F | Type 4 | walking | yes | grey | yes | no | yes | yes - early childood | N/A |
| F1.2 | *COL1A2*:c.1892G>T,p.Gly631Val | 8 years | Mixed South African | M | Type 4 | walking | yes | grey | yes | no | yes | yes - early childood | N/A |
| F1.3 | *COL1A2*:c.1892G>T,p.Gly631Val | 2 years | Mixed South African | M | Type 4 | walking | yes | grey | yes | no | yes | yes - early childood | N/A |
| F1.4 | *COL1A2*:c.1892G>T,p.Gly631Val | 12 years | Mixed South African | F | Type 4 | walking | yes | grey | yes | no | yes | yes - early childood | N/A |
| F1.5 | *COL1A2*:c.1892G>T,p.Gly631Val | 32 years | Mixed South African | F | Type 4 | walking | yes | grey | yes | no | yes | yes - early childood | N/A |
| F1.6 | *COL1A2*:c.1892G>T,p.Gly631Val | 6 years | Mixed South African | F | Type 4 | walking | yes | grey | yes | no | yes | yes - early childood | N/A |
| F2.1 | *COL1A2*:c.1892G>T,p.Gly631Val | 4 years | Mixed South African | F | Type 4 | walking | yes | grey | yes | no | yes | yes - early childood | N/A |
| F2.2 | *COL1A2*:c.1892G>T,p.Gly631Val | 15 years | Mixed South African | F | Type 4 | walking | yes | grey | yes | no | yes | yes - early childood | N/A |
| F2.3 | *COL1A2*:c.1892G>T,p.Gly631Val | 22 years | Mixed South African | F | Type 4 | walking | yes | grey | yes | no | yes | yes - early childood | N/A |
| F2.4 | *COL1A2*:c.1892G>T,p.Gly631Val | 39 years | Mixed South African | F | Type 4 | walking | yes | grey | yes | no | yes | yes - early childood | N/A |
| F2.5 | *COL1A2*:c.1892G>T,p.Gly631Val | 49 years | Mixed South African | F | Type 4 | walking | yes | grey | yes | no | yes | yes - early childood | N/A |
| F2.6 | *COL1A2*:c.1892G>T,p.Gly631Val | 20 years | Mixed South African | F | Type 4 | walking | yes | grey | yes | no | yes | yes - early childood | N/A |
| F2.7 | *COL1A2*:c.1892G>T,p.Gly631Val | 18 years | Mixed South African | F | Type 4 | walking | yes | grey | yes | no | yes | yes - early childood | N/A |
| F2.8 | *COL1A2*:c.1892G>T,p.Gly631Val | 3 years | Mixed South African | F | Type 4 | walking | yes | grey | yes | no | yes | yes - early childood | N/A |
| F2.9 | *COL1A2*:c.1892G>T,p.Gly631Val | 24 years | Mixed South African | F | Type 4 | walking | yes | grey | yes | no | yes | yes - early childood | N/A |
| F3.1 | *COL1A2*:c.1892G>T,p.Gly631Val | 39 years | Mixed South African | F | Type 4 | walking | yes | grey | yes | no | yes | yes - early childood | N/A |
| F3.2 | *COL1A2*:c.1892G>T,p.Gly631Val | 31 years | Mixed South African | F | Type 4 | walking | yes | grey | yes | no | yes | yes - early childood | N/A |
| F3.3 | *COL1A2*:c.1892G>T,p.Gly631Val | 7 years | Mixed South African | F | Type 4 | walking | yes | grey | yes | no | yes | yes - early childood | N/A |
| F3.4 | *COL1A2*:c.1892G>T,p.Gly631Val | 13 years | Mixed South African | M | Type 4 | walking | yes | grey | yes | no | yes | yes - early childood | N/A |
| F3.5 | *COL1A2*:c.1892G>T,p.Gly631Val | 36 years | Mixed South African | F | Type 4 | walking | yes | grey | yes | no | yes | yes - early childood | N/A |
| F3.6 | *COL1A2*:c.1892G>T,p.Gly631Val | 2 years | Mixed South African | M | Type 4 | walking | yes | grey | yes | no | yes | yes - early childood | N/A |
| F3.7 | *COL1A2*:c.1892G>T,p.Gly631Val | 23 years | Mixed South African | M | Type 4 | walking | yes | grey | yes | no | yes | yes - early childood | N/A |
| F3.8 | *COL1A2*:c.1892G>T,p.Gly631Val | 2 years | Mixed South African | F | Type 4 | walking | yes | grey | yes | no | yes | yes - early childood | N/A |
| F3.9 | *COL1A2*:c.1892G>T,p.Gly631Val | 13 years | Mixed South African | F | Type 5 | walking | yes | grey | yes | no | yes | yes - early childood | N/A |
| F3.10 | *COL1A2*:c.1892G>T,p.Gly631Val | 3 months | Mixed South African | M | Type 6 | N/A | yes | grey | N/A | no | yes | yes - prenatal onset | N/A |
| P1 | *COL1A1*:c.458_459insN[72],p.Gly16Leu155insN[24](*) | 32 years | Mixed South African | F | Type 4 | walking | yes | grey | yes | no | yes | yes | N/A |
| P2 | *COL1A1*:c.4163T>C,p.Leu1388Pro | 11 months | Mixed South African | M | Type 4 | walking | yes | grey | yes | no | yes | yes - prenatal onset | N/A |
| P3 | *COL1A2*:c.1459G>A,p.Gly487Arg | 2 months | Black South African | M | Type 3 | N/A | yes | blue | yes | no | yes | yes - prenatal onset | N/A |
| P4 | *COL1A2*:c.2215G>A,p.Gly739Arg | stillborn | Mixed South African | F | Type 2 | N/A | yes | blue | yes | N/A | yes | yes – multiple in-utero | N/A |
| P5 | *COL1A2*:c.2341G>C,p.Gly781Arg | 4 years | White South African | M | Type 4 | walking | yes | blue | yes | no | yes | yes | N/A |
| P6 | *COL1A2*:c.767G>A,p.Gly256Asp | 8 years | Black South African | F | Type 3 | walking - with assistive device | yes | blue | yes | no | yes | yes | N/A |
| P7 | *COL1A1*:c.458_459insN[72],p.Gly16Leu155insN[24](*) | 1 month | Mixed South African | F | Type 4 | N/A | yes | grey | yes | no | yes | yes - prenatal onset | N/A |
| P8 | *COL1A2*:c.1028G>A,p.Gly343Glu(*) | 20 years | White South African | F | Type 3 | wheelchair-bound | yes | blue | yes | no | yes | yes | N/A |
| P9 | *COL1A1*:c.750+1G>T(*) | 15 years | Black South African | F | Type 3 | wheelchair-bound | yes | blue | yes | no | yes | yes | N/A |
| P10 | *COL1A2*:c.2314G>A,p.Gly772Ser | 15 years | White South African | M | Type 4 | walking | yes | grey | yes | no | yes | yes | N/A |
| P11 | *COL1A2*:c.2314G>C,p.Gly772Arg | 5 years | Mixed South African | M | Type 4 | walking | yes | blue | yes | no | yes | yes | N/A |
| P12 | *COL1A1*:c.2830-2A>C(*) | Stillborn | Mixed South African | F | Type 2 | N/A | yes | blue | yes | N/A | yes | yes | N/A |
| P13 | *FKBP10*:c.831dupC | 4 years | Mixed South African | M | Type 3 | wheelchair-bound | yes |  | yes | no | yes | yes | N/A |
| P14 | *FKBP10*:c.831dupC | 10 years | Black South African | M | Type 3 | walking - with assistive device | yes |  | yes | no | yes | yes | N/A |
| P15 | *COL1A2*: c.1892G>T,p.Gly631Val | 2 months | Mixed South African | F | Type 4 | N/A | yes |  | yes | no | yes | yes | N/A |

DI: dentinogenesis imperfecta; HL: hearing loss; F: female; M: male; N/A: not applicable
