# Supplementary material for: Genetic basis of osteogenesis imperfecta from a single tertiary centre in South Africa
Source: Eur J Hum Genet. 2023 Dec 15;32(10):1285–90. doi: 10.1038/s41431-023-01509-3 (PMC11499597; doi:10.1038/s41431-023-01509-3)

**Supplementary information**

**Genetic basis of osteogenesis imperfecta from a single tertiary centre in South Africa**

Kimberly Christine Coetzer ^1^, Ekkehard Zöllner^2^, Shahida Moosa^1,3^*

1. Division of Molecular Biology and Human Genetics, Stellenbosch University Faculty of Medicine and Health Sciences, Tygerberg, 7505, Cape Town, South Africa
2. Department of Paediatrics, Stellenbosch University Faculty of Medicine and Health Sciences, Tygerberg, 7505, Cape Town, South Africa
3. Medical Genetics, Tygerberg Hospital, Tygerberg, 7505, Cape Town, South Africa

*Corresponding author:

Prof. Shahida Moosa

Division of Molecular Biology and Human Genetics

Stellenbosch University

Faculty of Medicine and Health Sciences

BMRI 1027

Francie Van Zijl Drive

Tygerberg 7505

Cape Town

South Africa

Tel: +27-21-9389218

Email: shahidamoosa@sun.ac.za

**Contents**

**Methods and materials:**

DNA extraction from blood and saliva samples.

**Supplementary Figures:**

**Figure S1.** Pathogenicity distribution of OI patients.

**Figure S2.** Gene distribution of OI patients.

**Supplementary 1: Methods and materials**

**DNA extractions from blood and saliva samples**

DNA was extracted from each sample using either the Qiagen Blood Mini Kit (Qiagen, Hilden, Germany) for blood samples or a standard saliva extraction protocol that uses PrepIt.LP2 (DNAgenotek) for saliva samples. Samples were extracted according to the manufacturer’s guidelines. In brief, for the saliva samples, the Oragene vials containing the saliva were inverted for 10 seconds before being incubated overnight at 50℃. Saliva (500ul) was transferred to a 1.5ml microcentrifuge tube and the remainder of the sample was transferred to a 2ml microcentrifuge tube and stored at -20°C for future use. Prepit·L2P (20ul) was added and mixed by inversion before vortexing for five seconds. The samples were incubated on ice for ten minutes. Samples were then spun at room temperature (RT) for 5 minutes at 15 000 X g. The clear supernatant was pipetted into a clean 1.5ml microcentrifuge tube and 99.9% cold EtOH (600ul) was added. After gentle inversion, samples were left to precipitate at RT for 10 minutes. Samples were then centrifuged for 2 minutes at RT at 15 000 X g. The supernatant was discarded and 70% EtOH (250ul) was added to the pellet for 1 minute at RT. Excess EtOH was removed and the samples were left to air dry at RT for two hours. The pellets were then dissolved in 100 μl of TE (pre-warmed @ 65℃) before being incubated on a rotator overnight at 4 ℃.

Protease (20ul) was added to a 1.5ml microcentrifuge tube with 200ul of blood for the blood samples. Buffer AL (200ul) was added and vortexed for 15 seconds before being incubated at 56℃ for 10 minutes. Samples were briefly centrifuged and 99.9% EtOH (200ul) was added to the sample before vortexing for 15 seconds. Samples were briefly centrifuged again and then added to the Qiagen Mini Spin Column without wetting the rim. The samples were centrifuged at 6000 X g for 1 minute. The filtrate was discarded and Buffer AW1 (500ul) was added. The samples were centrifuged for 1 minute at 6000 x g. The filtrate was discarded again and Buffer AW2 (500ul) was added before centrifuging at full speed for three minutes. The filtrate was removed, and the column was placed in a new collection tube. Buffer AE (50ul) was added to the column and left to incubate at room temperature for five minutes. Samples were centrifuged for 1 minute at 6000 x g and this elution step was repeated one more time. Samples were stored overnight at 4°C.


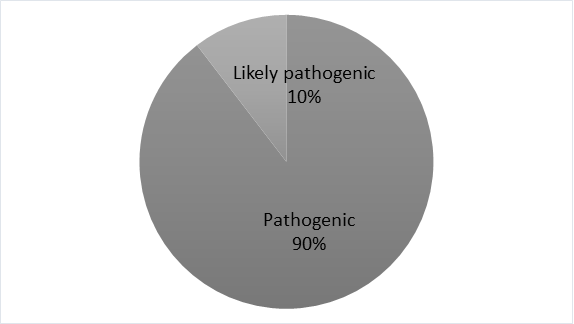


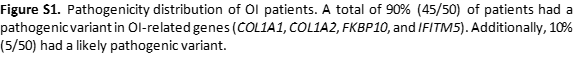


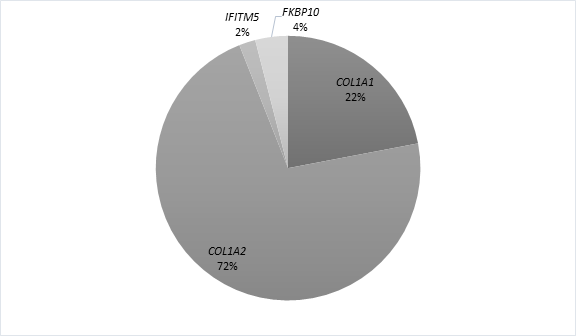


 
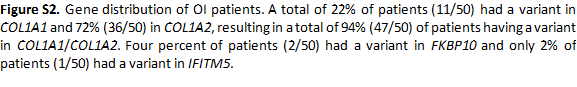

Supplement: Supplementary file 2 — Supplementary information [file 41431_2023_1509_MOESM2_ESM.docx]
